# Revitalizing *Pleurotus eryngii* polysaccharides: gamma irradiation boosts antidiabetic and antioxidant potential

**DOI:** 10.1186/s40643-025-00854-z

**Published:** 2025-05-23

**Authors:** Eman H. F. Abd El-Zaher, Ehab M. Tousson, Azza A. Mostafa, Enas M. El-Gaar, Galal Yahya, Yehia A.-G. Mahmoud

**Affiliations:** 1 Microbiology Unit, Botany Department, Faculty of Science, Tanta University, Tanta, Egypt; 2 Cell Biology and Histology, Zoology Department, Faculty of Science, Tanta University, Tanta, Egypt; 3 Biological and Environmental Department, Faculty of Home Economics, Al Azhar University, Tanta, Egypt; 4 Department of Microbiology and Immunology, Faculty of Pharmacy, Zagazig University, Zagazig, 44519 Egypt; 5 Molecular Biology Institute of Barcelona, Spanish National Research Council Catalonia, Barcelona, Spain

**Keywords:** Polysaccharides, Mushroom, Gamma radiation, Diabetic, Antioxidant, Histopathology

## Abstract

Polysaccharides derived from *Pleurotus eryngii* possess various bioactive properties, including antioxidant, antidiabetic, anti-inflammatory, and immunomodulatory effects. In this study, polysaccharides were extracted from *P. eryngii* fruiting bodies and exposed to gamma irradiation at doses of 50 and 100 kGy, with a dose rate of 5 kGy/h. The surface morphology of the polysaccharide irradiated at 100 kGy exhibited numerous pores and a smaller flake structure compared to those irradiated at 50 kGy and the non-irradiated sample. ^1^H and ^13^C NMR spectra of all samples indicated that both irradiated and non-irradiated polysaccharides exhibited α- and β-configurations, with signals corresponding to C1–C5 clearly observed. HPLC analysis of the polysaccharides revealed that glucose (75.23%), galactose (4.96%), glucuronic acid (1.38%), ribose (0.94%), rhamnose (2.35%), and mannose (3.87%) are the main components. All polysaccharides demonstrated antioxidant activity, which increased with concentration. Both non-irradiated and irradiated polysaccharides exhibited antidiabetic effects, significantly reducing blood glucose levels, and restoring insulin level with superiority of irradiated polysaccharides. Additionally, they significantly elevated body weight, slightly reduced MDA levels, and markedly enhanced catalase activity in treated rats compared to diabetic controls. The antidiabetic effects of the polysaccharides were further confirmed by histopathological examination of the pancreas and liver sections from polysaccharide-treated diabetic rats. This suggests that irradiation, by reducing the molecular weight of polysaccharides, enhances their bioavailability and efficacy in modulating glucose metabolism.

**Graphical Abstract:**

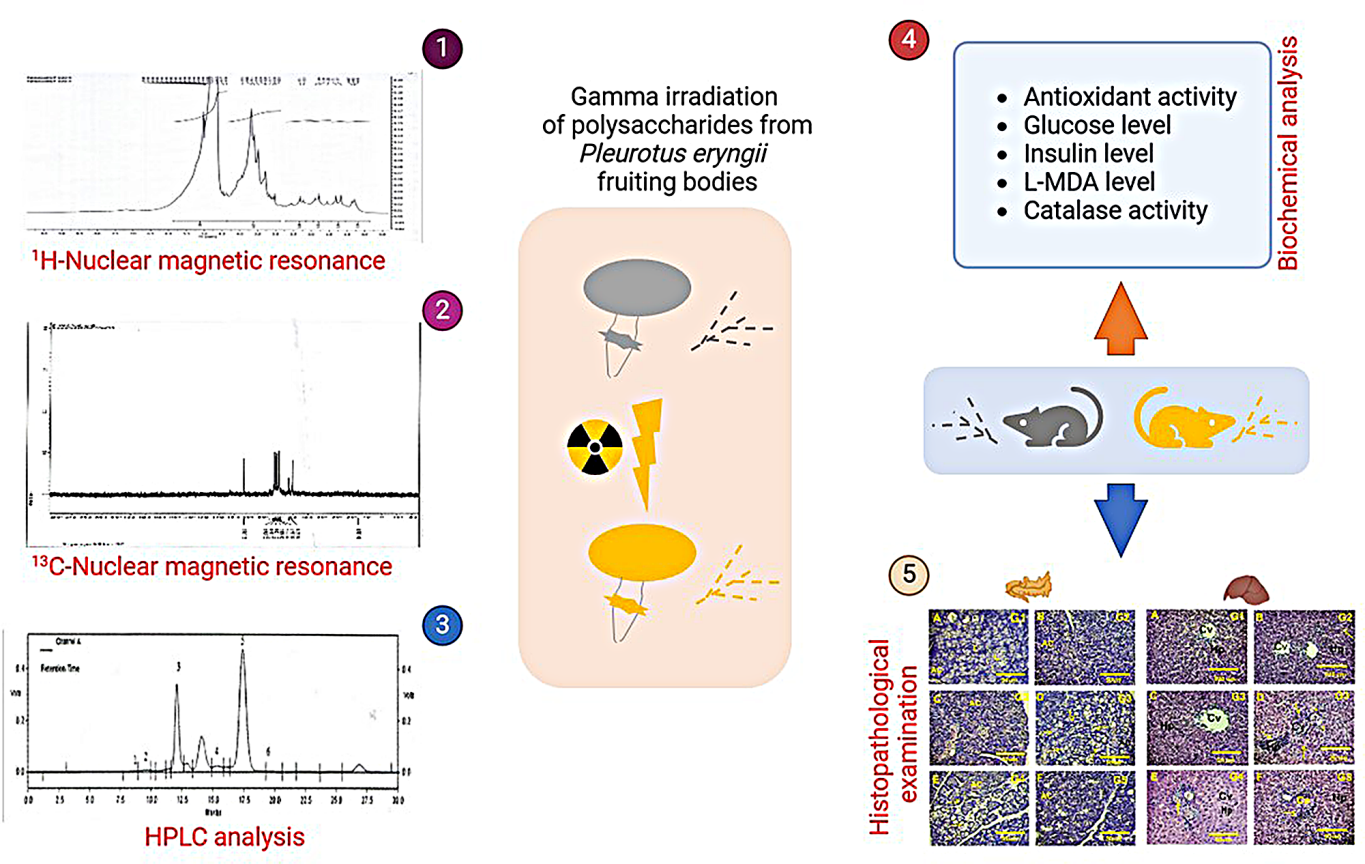

## Introduction

Diabetes mellitus (DM), a chronic metabolic disorder, has become a significant global health concern, imposing considerable strain on healthcare systems due to its substantial risks to large populations (Sugandh et al. 2023). The morbidity rate of DM has risen sharply in recent decades. According to the International Diabetes Federation survey (2021), there were 536.6 million people (aged 20–79 years) with diabetes worldwide, a number expected to reach 783.2 million by 2045 (Dong et al. 2023). Diabetes arises from insufficient insulin production or impaired insulin function, leading to elevated blood glucose levels (Sugandh et al. 2023). It also has detrimental effects on numerous organs, including the eyes, kidneys, nerves, heart, and blood vessels, representing a major burden on healthcare systems (Dong et al. 2023).

Commonly used chemical oral drugs and biological injections, such as biguanides, sulfonylureas, thiazolidinediones, meglitinides, acarbose, insulin and its analogs, α-glucosidase inhibitors, and dipeptidyl peptidase-4 inhibitors, are associated with noticeable side effects, particularly in the gastrointestinal tract (e.g., abdominal pain, cramps, flatulence, bloating, and diarrhea), as well as tolerability issues (Dong et al. 2023). Due to the harmful effects of chemical drugs, recent research has focused on discovering biological DM treatments from natural sources with fewer side effects. Natural resources, especially polysaccharides, have gained increased attention in treating DM in recent years (Kumari et al. 2023). Natural polysaccharides, characterized by lower toxicity and fewer side effects, are primarily extracted from the cell walls and membranes of bacteria, plants, and fungi (Xue et al. 2023).

Globally, approximately 2.2–3.8 million fungal species have been identified, with 150,000 species described, 2,000 recorded as edible, and over 200 species of wild mushrooms exhibiting therapeutic activity (Łysakowska et al. 2023). Mushrooms have been a part of the human diet for thousands of years due to their characteristic flavor and texture. In addition to their culinary appeal, mushrooms are widely used in medicinal fields (Liu et al. 2022). *P. eurotus* mushrooms are particularly valued for their low fat, high fiber, and high protein content (Venkata Krishna et al. 2023).

For health benefits, *P. eurotus* mushrooms have garnered more attention than other nutraceutical sources (Kumar et al. 2021). Polysaccharides are the primary bioactive components of some mushroom species, contributing significantly to their biological activity (Meera 2023). *P. eryngii*, a species of *P. ostreatus*, is an edible mushroom widely cultivated worldwide, known for its high nutritional value. It contains various active ingredients such as polysaccharides, proteins, minerals, trace elements, vitamins, terpenoids, peptides, and other bioactive compounds. *P. eryngii* polysaccharides exhibit numerous biological activities, including antitumor, antioxidant, hepatoprotective, anti-hyperlipidemic, and immune-boosting effects (Gong et al. 2022b; Dedousi et al. 2023; Petraglia et al. 2023). Mushroom polysaccharides are macromolecules composed of monosaccharide residues linked by glycosidic bonds, including glucans (glucose polymers) and heteroglycans (arabinose, mannose, fucose, galactose) (Guo et al. 2023).

However, high molecular weight polysaccharides are associated with high viscosity, which poses challenges for their use (Xu et al. 2022). Some researchers argue that only low molecular weight substances exhibit biological activity, as they are more easily absorbed by the body than high molecular weight polysaccharides (Xiong et al. 2020). Various methods have been employed to degrade polysaccharides, with chemical methods being time-consuming, expensive, and complex. In contrast, physical modifications, such as gamma irradiation, offer a low-cost, fast, and environmentally friendly alternative, characterized by high stability and the absence of chemical reagents or additional equipment (Sofi et al. 2013; Xiong et al. 2020).

The objectives of our study are to extract and characterize polysaccharides from *P. eryngii* and to investigate the effects of gamma irradiation on their molecular structure and surface morphology. Additionally, the study aims to evaluate the antioxidant and antidiabetic activities of both irradiated and non-irradiated polysaccharides, and to assess their biological effects on diabetic rats through biochemical and histopathological analyses.

## Methods/experimental

### Cultivation of *P. erngyii*

The *P. eryngii* mycelia C7 was obtained from Faculty of Agriculture, El-Azher University, was inoculated on Potato dextrose agar medium at 25–27 °C for a period of 10 days (Dedousi et al. 2023).

### Extraction and determination of polysaccharides from fruiting bodies of *P. eryngii*

The dried *P. eryngii* fruiting bodies were first ground using a mortar after being dried at 70 °C. The ground material was then boiled with distilled water (20 v/v) for 3 h, followed by centrifugation at 8,000 rpm for 30 min. The supernatant was deproteinized by adding Sevag’s reagent (chloroform: alcohol, 5:1) in equal volume. To precipitate the crude polysaccharides, 95% cold ethanol was added to the solution, which was then allowed to stand overnight at 4 °C. The solution was subsequently centrifuged again to collect the precipitate, which constituted the *P. eryngii* polysaccharides (Petraglia et al. 2023). The concentration of the extracted polysaccharides (partially purified by dialysis) was determined using the Phenol-Sulfuric acid method (Ellefsen et al. 2023), with the absorbance of the characteristic yellow-orange color measured at 490 nm.

### Radiation *P. eryngii* Endo polysaccharides

Gamma radiation was applied for extracted polysaccharide in closely capped tubes using a Cobalt-60 irradiator at Egyptian Atomic Energy Authority with a dose rate of 5 kGy/h at 50 and 100 kGy doses. The applied polysaccharide was stored at 4^◦^C until use (Xiong et al. 2020).

### Morphology observation of polysaccharides by scanning electron microscopy (SEM)

It was carried out at National Research Centre, Cairo using scanning electron microscope (Tescan vega3 SBU, Czech Republic) with 20KeV. It was mounted on aluminum microscopy stubs using carbon tape, then coated with a thin layer of gold for 120 s using Quroum technique Ltd, sputter coater (Q150t, England) (Xiong et al. 2020).

### High performance liquid chromatography (HPLC) analysis

Ten milligrams of the sample were hydrolyzed using a modified method from (Seedevi et al. 2019). The hydrolysis was carried out with 2 mL of 3 M trifluoroacetic acid at 95 °C for 8 h in a 10 mL sealed sample vial filled with nitrogen gas. After hydrolysis, the sample was cooled and centrifuged at 1,500 rpm for 5 min. The supernatant was transferred to a 5 mL micro-round flask, dried under reduced pressure, and then dissolved in 1 mL of ultrapure water. The resulting aqueous solution was filtered through a 0.45 μm membrane for HPLC analysis.

Sugar analysis in the filtrate was conducted using an HPLC system (Shimadzu Class-VPV5.03, Kyoto, Japan) equipped with a refractive index detector (RID-10 A Shimadzu), an LC-16ADVP binary pump, a DCou-14 A degasser, and a Shodex PL Hi-Plex Pb column. The mobile phase consisted of double-distilled water, with a flow rate of 1 mL/min. Peak identification was achieved by comparing retention times with standards. Glucose, galactose, glucuronic acid, ribose, mannose, and rhamnose standards were obtained from Sigma Chemicals. The analysis was performed at the National Research Centre, Cairo.

### Nuclear magnetic resonance (NMR) analysis

Polysaccharides sample (50 mg) were dissolved in 0.5 mL of deuterium (99%) and 30 µL acetone-d6 was added (Xiong et al. 2020). The ^1^H and ^13^C NMR spectra of polysaccharides were performed at 30^◦^C using a MH JOEOL-Japan at 500 MHz.

### Antioxidant activity of polysaccharides by 2,2 Di phenyl Picryl hydrocarbon (DPPH) radical scavenging activity

This method utilizes DPPH (2,2-diphenyl-1-picrylhydrazyl) as a stable free radical that changes color from purple to yellow upon scavenging. The degree of discoloration serves as an indicator of the hydrogen-donating ability of the compound. The antioxidant activity was estimated using the method of (Kim et al. 2006; Kayahan et al. 2023).

Various concentrations of irradiated and non-irradiated polysaccharides (0.625, 0.3125, 0.208, 0.156, and 0.125 mg/mL) were prepared and mixed with 3.9 mL of a DPPH solution (0.025 g/L in methanol). The mixture was shaken vigorously and allowed to react at room temperature, shielded from light, for 30 min. After the reaction period, the absorbance was measured at 515 nm using a spectrophotometer.

A blank was prepared by mixing 0.1 mL of methanol with 3.9 mL of DPPH solution. The percentage of DPPH scavenged was calculated using the following equation:

DPPH scavenging activity % = (A_o_ – A_s_/A_o_) * 100.

Where A_o_: the blank absorbance at 515 nm, A_s_: the sample absorbance at 515 nm.

### Hypoglycemic effect

Sixty male Wistar rats (weight: 180–210 g) were housed in polypropylene cages with bedding made from paddy husk (Balaji et al. 2020). The rats were acclimatized, provided with water and pelleted rat chow and maintained at a controlled temperature of 24 ± 2 °C with a 12-hour light/dark cycle for two weeks before the study. All procedures involving the rats were approved by the Institutional Animal Ethics Committee (IAEC) of Tanta University’s Faculty of Science.

Diabetes was induced in the rats using streptozotocin (STZ), which was dissolved in 100 mM citrate buffer (pH 4.5) at a dose of 40 mg/kg body weight and administered via intraperitoneal injection (Ghasemi and Jeddi 2023). To prevent hypoglycemic shock, a 10% glucose solution was provided to the STZ-injected rats 6 h after administration. After 21 h of monitoring, rats with blood glucose concentrations above 200 mg/dL were considered diabetic (Balaji et al. 2020).

The rats were divided into six groups as follows:

- Group I (CN): Control (normal rats).
- Group II (D): Diabetic rats (STZ, 40 mg/kg).
- Group III (CN + 1): Normal rats + non-irradiated polysaccharides (100 mg/kg).
- Group IV (CN + 2): Normal rats + irradiated polysaccharides (100 mg/kg).
- Group V (D + 1): Diabetic rats + non-irradiated polysaccharides (100 mg/kg).
- Group VI (D + 2): Diabetic rats + irradiated polysaccharides (100 mg/kg).

After six weeks, the animals were fasted overnight and anesthetized with pentobarbitone sodium (60 mg/kg). Blood samples were collected in non-heparinized tubes, centrifuged to obtain serum, and used for biochemical analysis. For histopathological studies, the pancreas and liver were excised and preserved in 10% formalin. Additionally, 10% (w/v) liver and kidney samples were homogenized in 0.9% normal saline (Ramadan et al. 2023), centrifuged at 4 °C and 4,000 rpm for 20 min, and the supernatants were used for biochemical analysis.

For histopathological examination, small tissue sections from the pancreas and liver were fixed in buffered neutral formalin (10%) for two days. The tissues were then dehydrated in a series of ethanol solutions, embedded in paraffin, and sectioned at 5 μm thickness. The sections were deparaffinized, stained with Harris hematoxylin and eosin for 10 min, and washed in running water for 15 min. The stained sections were examined microscopically for histopathological changes (Bakaç et al. 2023; Salama et al. 2023).

### Biochemical analysis

Serum insulin levels were analyzed using the method described by (Hussein et al. 2022). Blood glucose levels were determined following the protocol of (Thaeir H. Hadees and Manal Saleh Mahdi 2023). Liver and kidney L-Malondialdehyde (L-MDA) levels were measured spectrophotometrically using the methods of (Yousif et al. 2022) and (Natarajan et al. 2023), respectively. The activity of the catalase enzyme was determined using the method described by (Hou et al. 2022).

## Results

### Physicochemical characteristics of irradiated and Non-Irradiated polysaccharides from *P. eryngii*

**Fig. 1:**
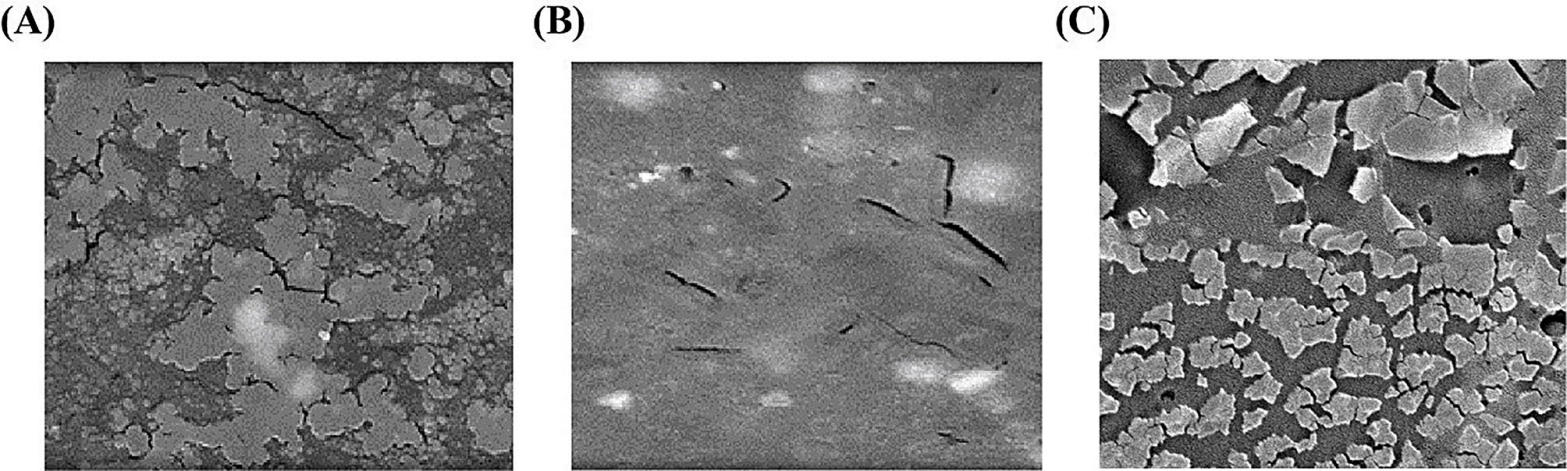
Scanning electron microscope for *P. eryngii* Polysaccharides. **(A)** Non-irradiated *P. eryngii* polysaccharides **(B)** Irradiated *P. eryngii* polysaccharides at 50 kGy. **(C)** Irradiated *P. eryngii* polysaccharides at 100 kGy

The morphology of irradiated (50 kGy and 100 kGy) and non-irradiated polysaccharides from *P. eryngii* was assessed using microscopic analysis. Non-irradiated polysaccharides appeared as irregular flakes with a smooth surface (Fig. 1A). Upon gamma irradiation, the surface at 50 kGy displayed slight wrinkling and a sheet-like appearance (Fig. 1B). At 100 kGy, the polysaccharides exhibited smaller flake structures with numerous pores (Fig. 1C), consistent with the findings of (Xiong et al. 2020).

### Nuclear magnetic resonance (^1^H & ^13^C NMR), and High-Performance liquid chromatography (HPLC) analysis of polysaccharides obtained from *P. eryngii*

**Fig. 2:**
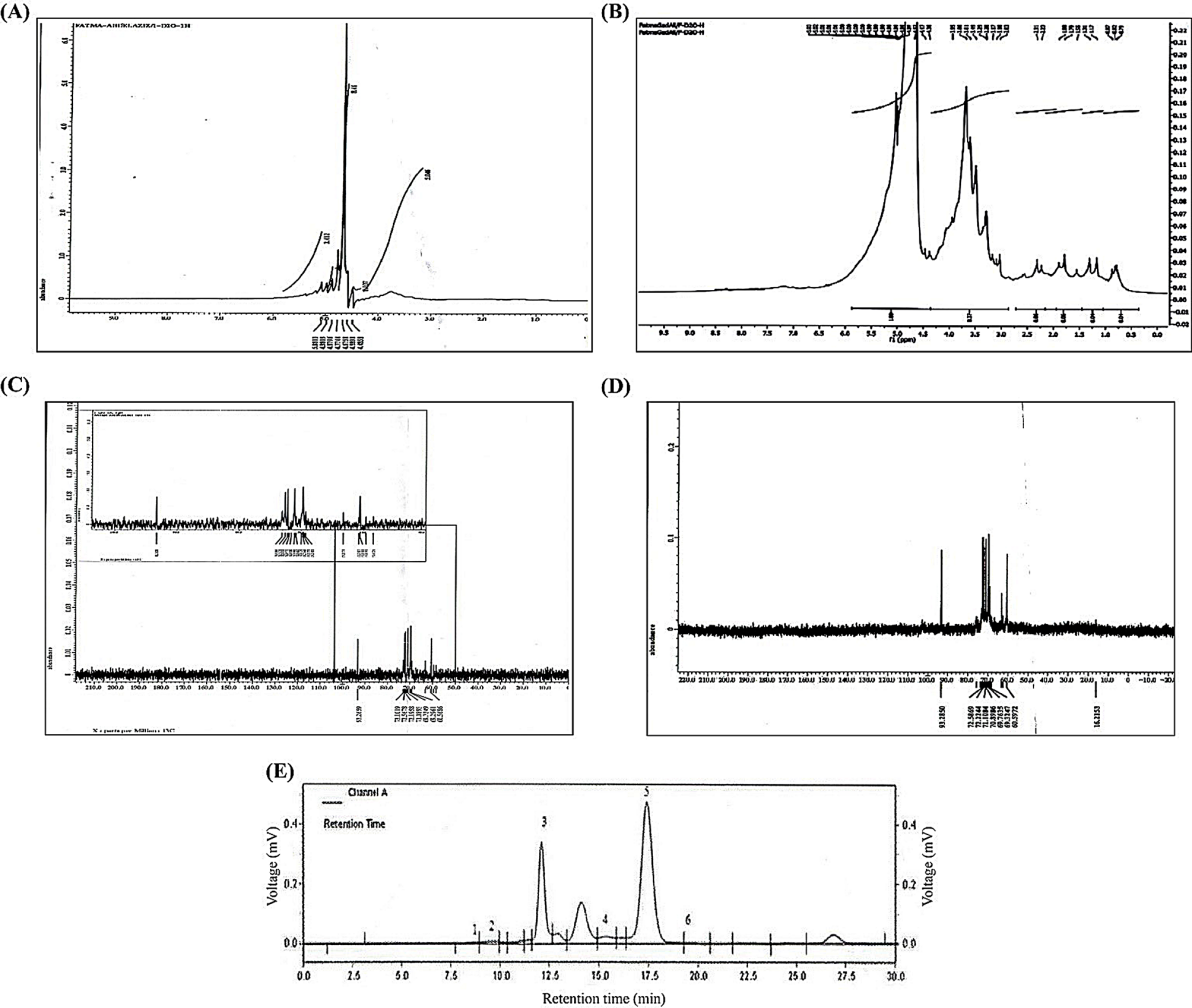
Nuclear magnetic resonance and HPLC analysis of polysaccharides obtained from *P. eryngii* and 100 kGy irradiated polysaccharides. **(A)** The ^1^H NMR analysis of non-irradiated polysaccharides obtained from *P. eryngii*. **(B)** The ^1^H NMR analysis of *P. eryngii* irradiated polysaccharides at 100 kGy dose. **(C)** The ^13^C NMR analysis of non-irradiated polysaccharides obtained from *P. eryngii.***(D)** The ^13^C NMR analysis of *P. eryngii* irradiated polysaccharides at 100 kGy dose. **(E)** HPLC analysis of monomer of polysaccharides obtained from *P. eryngii*. Peaks no 1, 2, 3, 4, 5 and 6 refer to mannose, ribose, rhamnose, glucuronic, glucose and galactose sugars respectively. (Figure 2A and C, and 2E are adapted and modified with permission from (Abd El-Zaher et al. 2022)

The ^1^H NMR spectrum of polysaccharides obtained from *P. eryngii* was shown in Fig. 2A. All signals were observed in the range of δ 3.5–5 ppm, indicating the presence of the α-configuration. The signals of non-irradiated polysaccharides appeared around δ 5.0 ppm, confirming the presence of both α- and β-configurations. The main signals at δ 4.4–4.8 ppm suggest that the main chain predominantly adopts a β-configuration.

The ^1^H NMR spectrum of irradiated polysaccharides was shown in Fig. 2B. All the signals were in the range δ 0.5–5.5 ppm which indicated presence α-configuration, while vibration of β-configuration was at δ 4.5–4.8 ppm. The signals of irradiated polysaccharides were around δ 3.5 and 5.0 ppm which ensure possessing α and β configuration. The main signals were at δ 4.5–4.8 ppm indicating β- configuration. Presence of more than one main signal indicated that radiation possibly degraded the glycoside bond between glucose units of Polysaccharides.

The ^13^C NMR spectrum of polysaccharides was shown in Fig. 2C. The peaks at 93.26, 71.08, 73.10, 69.29 and 69.73 were the signals of C-1, C-2, C-3, C-4 and C-5 respectively.

The ^13^C NMR spectrum of irradiated Polysaccharides was shown in Fig. 2D. The peaks at 93.28, 71.10, 72.5, 69.32 and 70.89 were the signals of C-1, C-2, C-3, C-4 and C-5 respectively. Signal at 5.28–4.41 ppm was the polysaccharides terminal hydrogen. In the ^13^C NMR spectrum, there were three main terminal carbon signals between 95 and 105 ppm, including 102.9, 97.8 and 93.2 ppm. Signal at 75–68 ppm was specialized to the signal of C–O, generally the C-2 to C-5 signal. Moreover, δ 93.2 ppm, 5.06 ppm could be corresponded to α-D-glucan. Collectively, spectroscopic analysis indicates that gamma irradiation did not alter the overall structure of these polysaccharides, consistent with findings from (Xiong et al. 2020). Comparisons with other studies showed that similar compositions were reported for polysaccharides from different mushroom species, emphasizing the high glucose content.

HPLC analysis revealed that polysaccharides from *P. eryngii* were composed of glucose, galactose, glucuronic acid, ribose, rhamnose, and mannose, with concentrations of 75.23%, 4.96%, 1.38%, 0.94%, 2.35%, and 3.87%, respectively (Fig. 2E).

### Antioxidant activity Di phenyl Picryl hydrocarbon (DPPH) radical scavenging activity assay

**Fig. 3:**
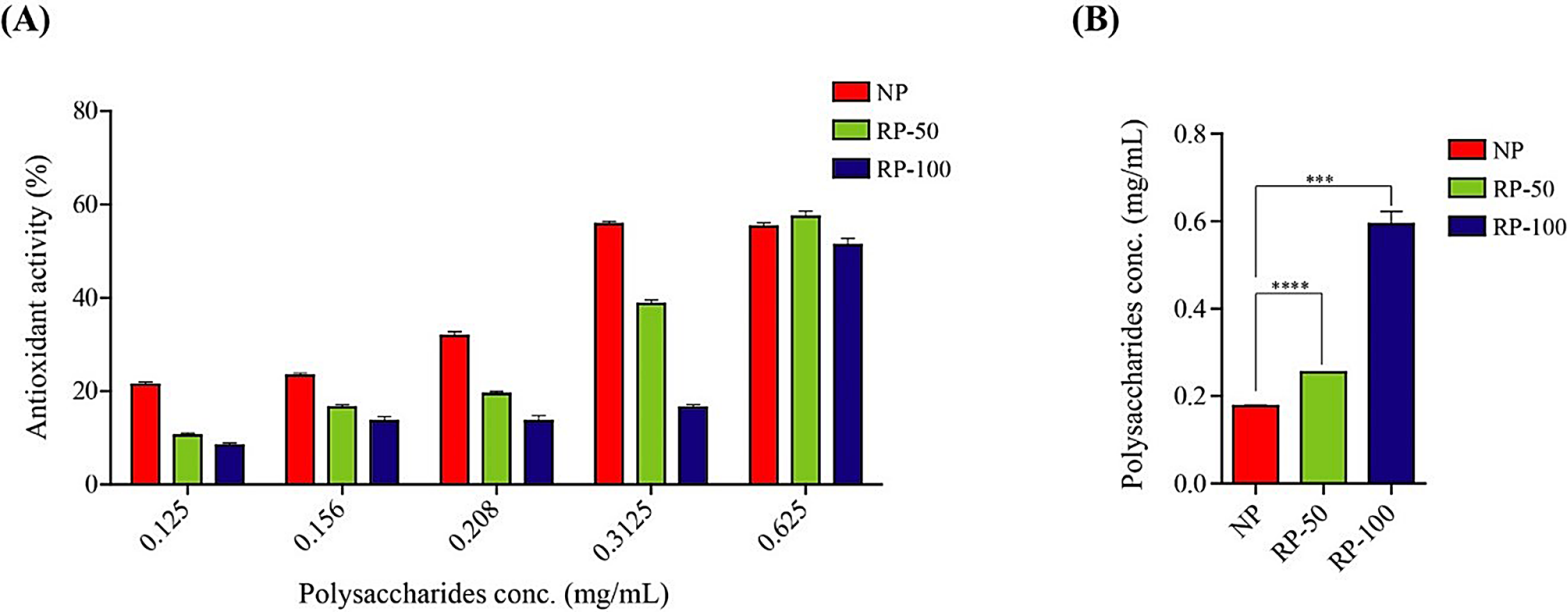
Antioxidant activity of non-irradiated and irradiated polysaccharides (50 and 100 kGy) from *P. eryngii* at different doses. **(A)** DPPH assay for estimating the antioxidant activity. **(B)** IC50 of antioxidant activity of non-irradiated polysaccharides and irradiated polysaccharides from *P. eryngii* by DPPH. *** *p* < 0.001, **** *p* < 0.0001. Statistical significance was analyzed by Two Tailed Unpaired T test

As illustrated in Fig. 3A, the DPPH radical scavenging activity of both non-irradiated and irradiated polysaccharides increased progressively with higher concentrations. Among the irradiated polysaccharides, those treated at 50 kGy demonstrated better scavenging activity at a concentration of 0.625 mg/mL compared to the non-irradiated polysaccharides, with irradiated polysaccharides at 100 kGy showing slightly lower activity. At a concentration of 0.625 mg/mL, the scavenging activities of all samples were closely comparable. However, when assessing the half-maximal inhibitory concentration (IC50), non-irradiated polysaccharides exhibited a lower IC50 (0.1765 ± 0.002 mg/mL), indicating superior in vitro scavenging efficiency compared to 50 kGy irradiated polysaccharides (0.2546 ± 0.001 mg/mL) and irradiated polysaccharides at 100 kGy (IC50 of 0.5931 ± 0.0297 mg/mL) as shown in Fig. 3B.

### Hypoglycemic effect of non-irradiated and irradiated polysaccharides from *P. eryngii* on diabetic Wistar rats

**Fig. 4:**
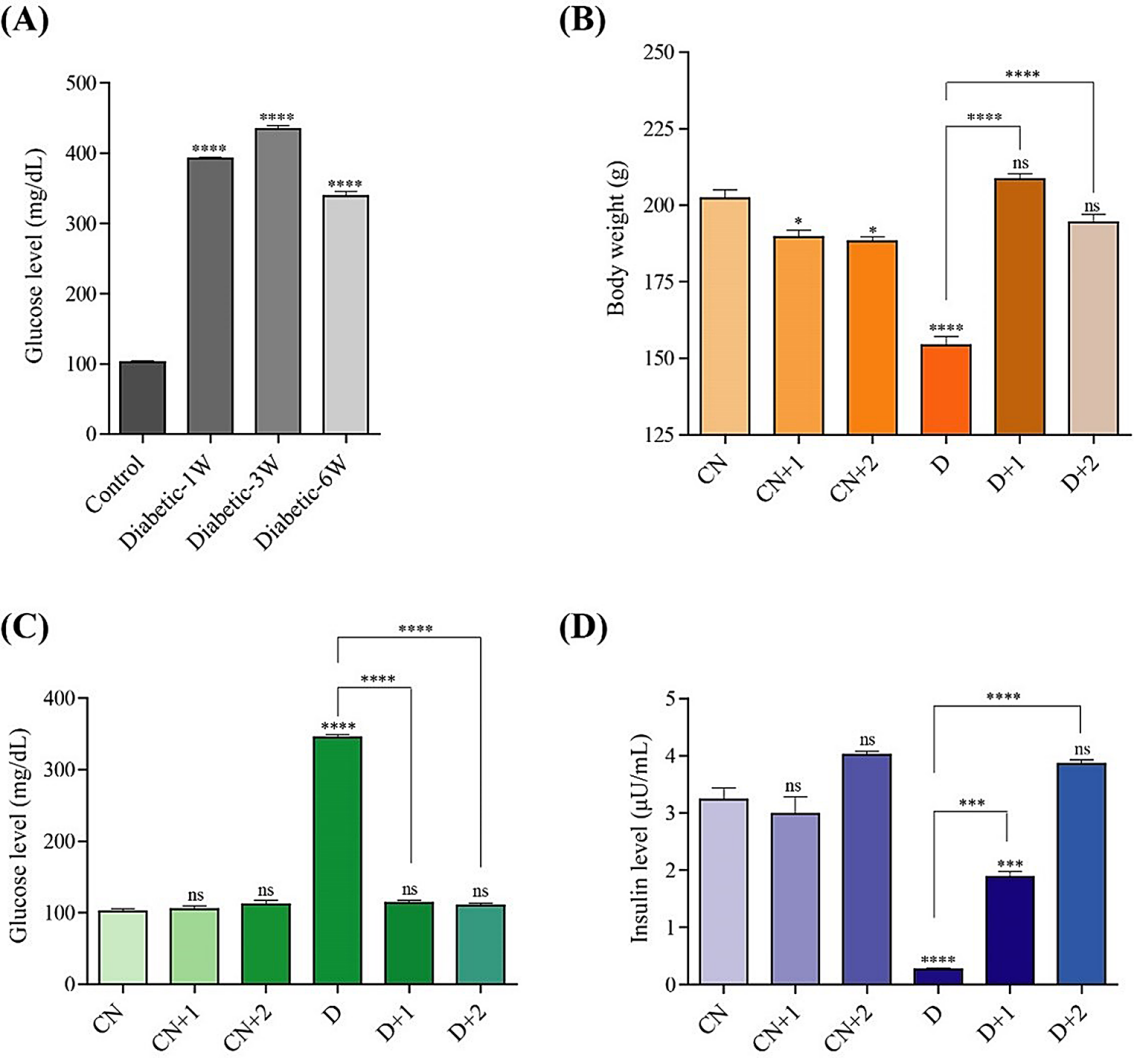
Metabolic effects of irradiated polysaccharides in normal and STZ- induced diabetic rats. **(A)** Serum glucose concentration in normal and STZ-induced diabetic rat after 1, 3 and 6 weeks. **(B)** Changes in the mean body weight between the different groups under study. **(C)** Serum glucose concentration in different treated groups. **(D)** Serum insulin level in different treated groups. ns (no significance), * *p* < 0.05. ** *p* < 0.01, *** *p* < 0.001, **** *p* < 0.0001. Statistical significance was analyzed by One-way ANOVA followed by Bonferroni’s multiple comparison test

After 1, 3, and 6 weeks of the experiment, a significant increase in serum glucose levels was observed in streptozotocin (STZ)-induced diabetic rats compared to the control group. The diabetic rats showed a significant elevation in serum glucose levels at all time points compared to the control group. Although there is a slight decrease in glucose levels at 6 weeks compared to earlier time points, the levels remain significantly elevated (Fig. 4A).

Diabetic rats showed a significant decrease in body weight compared to the control group, in line with previous findings (El Barky et al. 2018). Both non-irradiated and irradiated polysaccharides improved the body weight of diabetic rats, indicating a significant recovery effect (Fig. 4B). Lower molecular weight and viscosity of irradiated polysaccharides might have contributed to better biological activity.

#### Serum glucose level

Treatment with 100 mg/kg body weight of both non-irradiated polysaccharides and irradiated polysaccharides (100 kGy dose) from *P. eryngii* led to a significant reduction in elevated serum glucose levels in STZ-induced diabetic rats after six weeks, as compared to the untreated diabetic control group (Fig. 4C).

Additionally, the oral administration of non-irradiated and irradiated polysaccharides from *P. eryngii* to normal rats resulted in no significant change in serum glucose levels compared to the normal control group at the end of the experiment.

#### Insulin level (µU/mL)

As shown in Fig. 6, Diabetic rats (Group D) displayed significantly reduced insulin levels compared to the control group. Treatment with non-irradiated polysaccharides (D + 1) resulted in a significant increase in insulin levels, although these levels remained lower than the control. Diabetic rats treated with irradiated polysaccharides (D + 2) showed insulin levels comparable to the control group. The insulin level in diabetic rats treated with irradiated polysaccharides did not differ significantly from that of the normal control groups, as shown in Fig. 4D.

### Determination of liver and kidney L-Malondialdehyde (L- MDA)

**Fig. 5:**
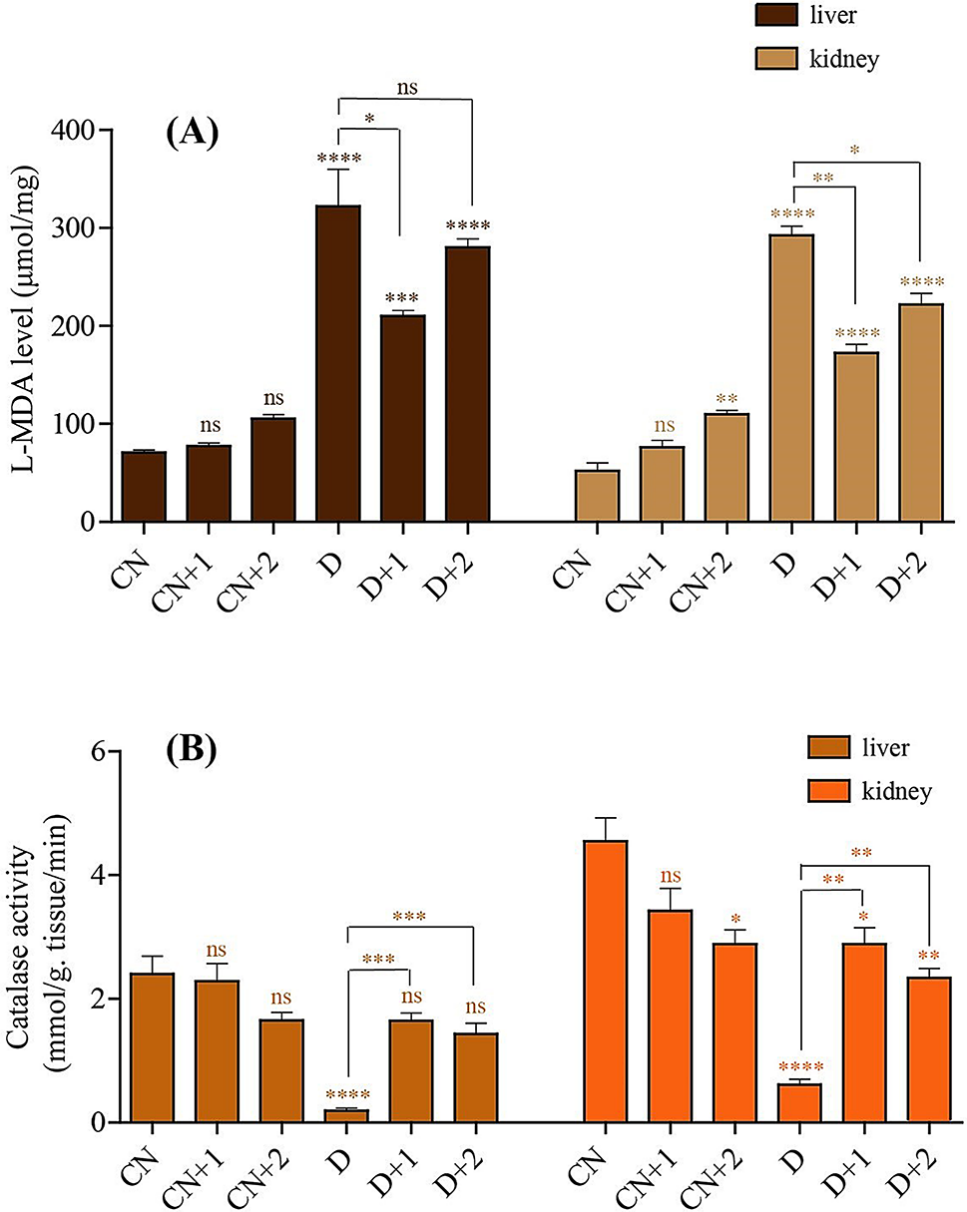
Biochemical impact of irradiated polysaccharides on oxidative stress in liver and kidney tissues. **(A)** L- MDA level of liver and kidney tissues in different treated groups. **(B)** Catalase activity in liver and kidney tissues in different treated groups. ns (no significance), * *p* < 0.05. ** *p* < 0.01, *** *p* < 0.001, **** *p* < 0.0001. Statistical significance was analyzed by One-way ANOVA followed by Bonferroni’s multiple comparison test

In liver tissue, the level of lipid peroxidation marker L-MDA was significantly elevated in diabetic rats compared to the control group, as shown in Fig. 5A. There was no significant difference between the L-MDA levels in normal rats fed with either type of polysaccharide and the control group. While diabetic rats that were fed on non-irradiated or irradiated polysaccharides displayed lower L-MDA levels that were significant in case of non-irradiated polysaccharides but non-significant for irradiated polysaccharides. In kidney tissue, the diabetic groups fed with non-irradiated or irradiated polysaccharides showed a significant decrease in L-MDA levels compared to diabetic control group as shown in Fig. 5A.

### Determination of liver and kidney catalase

Diabetic rats appeared a significantly low catalase enzyme activity of liver homogenate tissues as compared to control rats. Feeding diabetic rats with non-irradiated or irradiative polysaccharides recorded significantly decreased of enzyme activity than control group as was presented in Fig. 5B. Catalase enzyme activity of kidney homogenate tissues for all tested animals recorded significant increased in all tested groups as compared to diabetic induced rats. Oral administration of normal rats and diabetic rats of two polysaccharides had significant declination in catalase activity than control rats as shown in Fig. 5B.

### Histopathological results

#### The pancreas histopathology

**Fig. 6:**
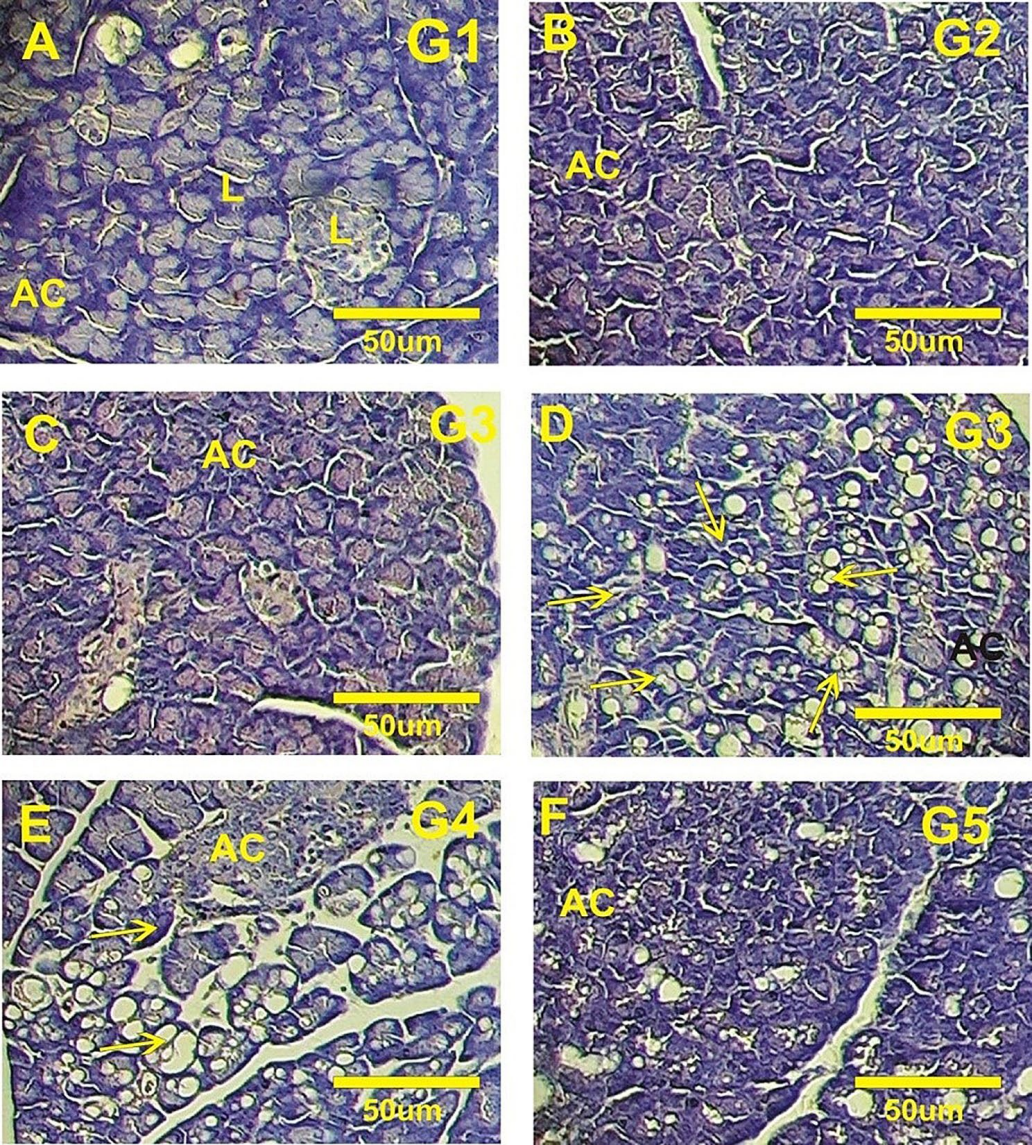
Photomicrograph of pancreatic tissue stained with hematoxylin and eosin. **(A)** Control Wistar rats. **(B)** Normal rats orally feeding with non-irradiated polysaccharides. **(C)** Normal rats orally feeding with irradiated polysaccharides. **(D)** Diabetic rats. **(E)** Treated diabetic rats with non-irradiated polysaccharides. **(F)** Treated diabetic rats with irradiative polysaccharides. Acinar cells (AC), Langerhans’ islet (L)

In the study, normal rats that were orally administered either non-irradiated or irradiated polysaccharides displayed a normal pancreatic structure. This included pancreatic acini with amphophilic cytoplasm and basal nuclei (Fig. 6A-C). The Langerhans islets appeared normal, with a regular round shape, numerous β-cells featuring well-defined, spherical nuclei, and clearly distinguishable connective tissue contributing to the islets’ characteristic round form. In contrast, diabetic rats exhibited reduced islet size, a lower number of β-cells, and degeneration in the connective tissue surrounding the islets, resulting in an irregular islet shape (Fig. 6D).

Diabetic rats treated with non-irradiated polysaccharides showed substantial improvement, with numerous islets returning to a nearly normal shape and exhibiting regenerated β-cells (Fig. 6E). On the other hand, diabetic rats treated with irradiated polysaccharides displayed partial amelioration, with some Langerhans islet cells showing nearly normal nuclei (Fig. 6F).

#### The liver histopathology

**Fig. 7:**
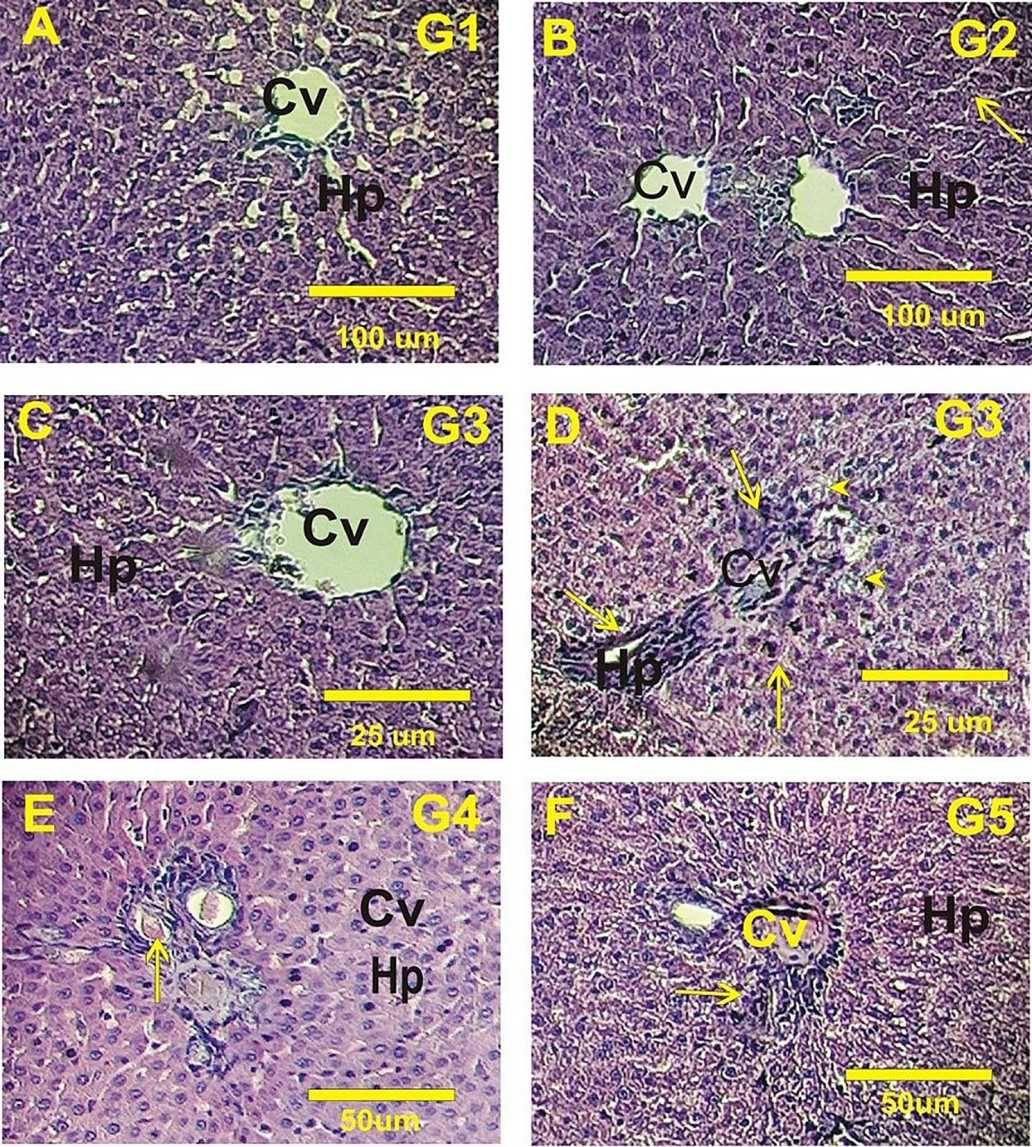
Hematoxylin and eosin-stained photomicrographs of liver tissue in Wistar rats. **(A)** Control Wistar rats showing normal hepatic structure. **(B)** Normal rats orally feeding with non-irradiated polysaccharides. **(C)** Normal rats orally feeding with irradiated polysaccharides. **(D)** Diabetic rats. **(E)** Treated diabetic rats with non-irradiated polysaccharides. **(F)** Treated diabetic rats with irradiative polysaccharides. Cv: central vein, Hp: Hepatocyte plates

In male Wistar rats, liver sections from control animals, as well as normal rats fed either non-irradiated or irradiated polysaccharides, displayed normal hepatocyte structures. These included polygonal-shaped hepatocytes with prominent, round nuclei, eosinophilic cytoplasm, and well-organized hepatic sinusoids between the hepatic cords, with Kupffer cells arranged neatly (Fig. 7A-C). In contrast, liver sections from diabetic rats showed significant hepatotoxicity. This was characterized by pronounced inflammation, degeneration of hepatic cords, numerous apoptotic cells, extensive diffuse necrosis, and congested blood sinusoids (Fig. 7D). Diabetic rats treated with non-irradiated polysaccharides exhibited mild to moderate inflammation and congestion in the blood sinusoids (Fig. 7E). Conversely, diabetic rats treated with irradiated polysaccharides showed notable improvement, with hepatocytes displaying only mild degeneration and a reduction in inflammatory cells (Fig. 7F).

## Discussion

This study evaluated the effects of gamma irradiation on the physicochemical properties, antioxidant activity, and hypoglycemic effects of polysaccharides extracted from *P. eryngii*. The findings demonstrated the preservation and enhancement of the biological activities of these polysaccharides following irradiation, paving the way for their potential applications in the food and pharmaceutical industries.

### Physicochemical properties of irradiated polysaccharides

Gamma irradiation at doses of 50 kGy and 100 kGy induced noticeable changes in the morphology of *P. eryngii* polysaccharides, as observed through surface structure alterations. Non-irradiated polysaccharides exhibited smooth, irregular flakes, while irradiation at 50 kGy resulted in a slightly wrinkled, sheet-like appearance. At 100 kGy, the polysaccharides showed a more fragmented structure with numerous pores. These morphological changes suggest that gamma irradiation leads to degradation of the polysaccharide chains, consistent with previous studies indicating that irradiation can cause significant molecular scission (Xiong et al. 2020). This degradation likely contributes to the observed decrease in molecular weight, enhancing solubility and potentially altering the biological activities of the polysaccharides.

The NMR spectra provided further insights into the structural modifications of the polysaccharides post-irradiation. The ^1^H NMR spectrum indicated that both irradiated and non-irradiated polysaccharides possessed α and β configurations, with the main signals corresponding to β-configuration. However, irradiation led to a broader range of chemical shifts and the emergence of additional peaks, particularly in the region corresponding to β-configuration, suggesting partial degradation of glycosidic bonds. The ^13^C NMR spectrum also showed slight shifts in the peaks of irradiated polysaccharides, which may be attributed to changes in the chemical environment of the carbon atoms due to irradiation-induced degradation. These findings align with previous studies, such as (Nakahara et al. 2020; Xiong et al. 2020), which also reported that gamma irradiation alters the structural integrity of polysaccharides.

### Impact on monosaccharide composition and antioxidant activity

HPLC analysis demonstrated that the monosaccharide composition of *P. eryngii* polysaccharides, predominantly glucose, galactose, glucuronic acid, ribose, rhamnose, and mannose, remained largely unchanged post-irradiation. This stability in monosaccharide composition suggests that while gamma irradiation affects the molecular weight and structure of polysaccharides, it does not significantly alter the basic monosaccharide units, which is consistent with findings by (Xiong et al. 2020) on other mushroom polysaccharides. This indicates that gamma irradiation may be a viable method for modifying polysaccharide properties without compromising their fundamental chemical makeup.

Regarding antioxidant activity, the DPPH radical scavenging assay revealed that both irradiated and non-irradiated polysaccharides exhibited dose-dependent antioxidant activities with superiority of non-irradiated polysaccharides. Interestingly, polysaccharides irradiated at 50 kGy exhibited comparable yet slightly lower scavenging activity at similar concentrations to non-irradiated samples, although the overall antioxidant potential declined with higher irradiation doses. The IC50 values indicated that non-irradiated polysaccharides had the strongest antioxidant effect, followed by 50 kGy -irradiated, then 100 kGy irradiated polysaccharides showing the lowest activity. These results suggest that while moderate gamma irradiation affects the antioxidant properties of polysaccharides, their antioxidant activity is still preserved, potentially due to the formation of smaller polysaccharide fragments with free radical scavenging capabilities, as also suggested by (Zhang et al. 2020). However, excessive irradiation might lead to over-degradation, diminishing these beneficial properties.

Zhang et al. examined *P. eryngii* polysaccharide for its antioxidant and found that acted as good antioxidant (Zhang et al. 2020). Xiong et al. showed that irradiated polysaccharides from *Morchella sextelata* at 1000 kGy dose had higher DPPH scavenging activity than other polysaccharides at low dose of gamma radiation than non-irradiated polysaccharides (Xiong et al. 2020). It was found that *P. ostreatus* polysaccharides with low molecular weight had the best antioxidant effect (Bai et al. 2022). Gong et al. stated distilled-water-eluting polysaccharide of *P. eryngii* exhibited better antioxidant than NaCl-eluted polysaccharide, probably due to higher molecular weight and β-glycosidic bond (Gong et al. 2022b).

Zhang et al. reported that two extracellular polysaccharides from *P. eryngii* SI-04 improved renal antioxidant status such as GSH-Px, CAT and MDA in streptozotocin -induced diabetic mice (Zhang et al. 2018). *Lentinus edodes* polysaccharides can reduce MDA levels in STZ induced diabetic mice (Gong et al. 2022a).

### Hypoglycemic effects and biological activities in diabetic rats

The hypoglycemic effects of *P. eryngii* polysaccharides were evaluated in streptozotocin-induced diabetic rats. Both non-irradiated and irradiated polysaccharides were effective in reducing serum glucose levels, improving insulin levels, and ameliorating body weight loss in diabetic rats, these results align with research by Ganesan and Xu and others on the hypoglycemic effects of mushroom polysaccharides (Ganesan and Xu 2019). Notably, irradiated polysaccharides at 100 kGy were particularly effective in normalizing insulin levels, bringing them closer to those observed in control rats. This suggests that irradiation, by reducing the molecular weight of polysaccharides, enhances their bioavailability and efficacy in modulating glucose metabolism, which agrees with findings by (Li et al. 2022). The ability of irradiated polysaccharides to elevate insulin levels and improve glucose uptake go in accordance to (Ganesan and Xu 2019) which highlights their potential as functional ingredients in managing diabetes.

Yang et al. appeared that body weights of STZ-induced diabetic rats, can be recovered by the hypoglycemic treatment (Yang et al. 2008). Diao et al. reported that STZ induced animals had lower body weight than the control rats and its body weight were increased by treatment with *Inonotus obliquus* polysaccharides (Diao et al. 2014). Zhang et al. demonstrated the body weight of diabetic mice was lost as compared to control group, it was increased through application of *P. djamor* polysaccharides as hypoglycemic treatment (Zhang et al. 2015). Body weights significantly increased when crude polysaccharides and rhamnose-enriched polysaccharides from *G. lithophila* were administered as hypoglycemic agents (Seedevi et al. 2020).

### Antioxidant enzyme activities and organ protection

The study also investigated the effect of polysaccharides on oxidative stress markers such as malondialdehyde (MDA) in liver and kidney tissues. Diabetic rats treated with polysaccharides showed reductions in MDA levels, indicating enhanced antioxidant defenses and reduced lipid peroxidation. This protective effect was more pronounced with non-irradiated polysaccharides, suggesting that gamma irradiation might affect the ability of polysaccharides to mitigate oxidative stress in diabetic conditions. The findings are in line with previous studies showing that polysaccharides can upregulate antioxidant enzymes and protect against oxidative damage in diabetic models (Gong et al. 2022b; Sun et al. 2022).

Balaji et al. stated that treatment with hot water extract from *P. pulmonarius* improved liver tissue by the proliferation of bile ducts and irregularities no longer existed in liver tissue (Balaji et al. 2020). Histological damage associated with diabetes was confirmed by a reduction in abnormal lipid accumulation and excessive hyperglycemia (Shao et al. 2014). Zhang et al. explained that treatment of diabetic rats with polysaccharides from *P. ostreatus* repaired pancreatic tissue where higher amount pancreatic beta cells was noticed and was uniformly distributed and a better islet structure (Zhang et al. 2016a, b). Additionally, lentinan an active ingredient purified from the bodies of *L. edodes* was effective in blocking the generation of reactive oxygen species, inhibiting JNK and p38 MAPK signaling pathways, and preventing NF-kB activation, which in turn halted pancreatic beta-cell apoptosis (Zhang et al. 2016c).

## Conclusion

Given the rising global prevalence of DM and its status as a major health threat, there is an immediate need to discover novel anti-diabetic compounds from natural sources. Recently, mushroom polysaccharides have been explored in the anti-diabetes field owing to their broad availability, low toxicity, structural diversity, and multiple bioactivities, making them promising candidates for functional foods and nutraceuticals. Polysaccharides from *P. eryngii*, fruiting bodies were exposed to gamma radiation at different doses. The ^1^H and ^13^C NMR spectra explained that irradiated and non-irradiated polysaccharides had α and β configuration and the signals of C1 - C5 were appeared. All polysaccharides possessed antioxidant activity. Polysaccharides (non-irradiated and irradiated at 100 kGy dose) had hypoglycemic effects, significantly reduced blood glucose level, and restored weight of diabetic rats, increasing insulin level than diabetic rats, reduced L-MDA level and significantly increased catalase activity. In liver and pancreas sections, polysaccharides exhibited beneficial effects in alleviating histological damage at the organ level.

## Data Availability

The data supporting this study’s findings are available from the corresponding author upon reasonable request.

## Abbreviations

^1^H NMR: Proton Nuclear Magnetic Resonance
^13^C NMR: Carbon-13 Nuclear Magnetic Resonance
Ac: Acinar Cells
CAT: Catalase
CV: Central Vein
DM: Diabetes Mellitus
DPPH: 2,2-Diphenyl-1-Picrylhydrazyl
GSH-Px: Glutathione Peroxidase
Hp: Hepatocyte Plates
HPLC: High-Performance Liquid Chromatography
IAEC: Institutional Animal Ethics Committee
IC50: Half-Maximal Inhibitory Concentration
kGy: Kilogray
L-MDA: L-Malondialdehyde
STZ: Streptozotocin
